# Supplementary material for: Post-Analysis of Predictive Modeling with an Epidemiological Example
Source: Healthcare (Basel). 2021 Jun 24;9(7):792. doi: 10.3390/healthcare9070792 (PMC8304882; doi:10.3390/healthcare9070792)
Supplement: Supplementary file 1 [file healthcare-09-00792-s001.zip › healthcare-1249590-supplementary.pdf]

Post-analysis of predictive modeling with an epidemiological example

Christina Brester[a], Ari Voutilainen[b], Tomi-Pekka Tuomainen[b], Jussi Kauhanen[b], Mikko Kolehmainen[a]

[a]Department of Environmental and Biological Sciences, University of Eastern Finland, Yliopistonranta 1 E, P.O. Box 1627, FI-70211 Kuopio, Finland;

[b]Institute of Public Health and Clinical Nutrition, University of Eastern Finland, Yliopistonranta 1 C, P.O. Box 1627, FI-70211 Kuopio, Finland

Corresponding author: Christina Brester, Yliopistonranta 1 E, P.O. Box 1627, FI-70211 Kuopio, Finland

+358449669095, kristina.brester@uef.fi, christina.brester@gmail.com

Supplementary Material

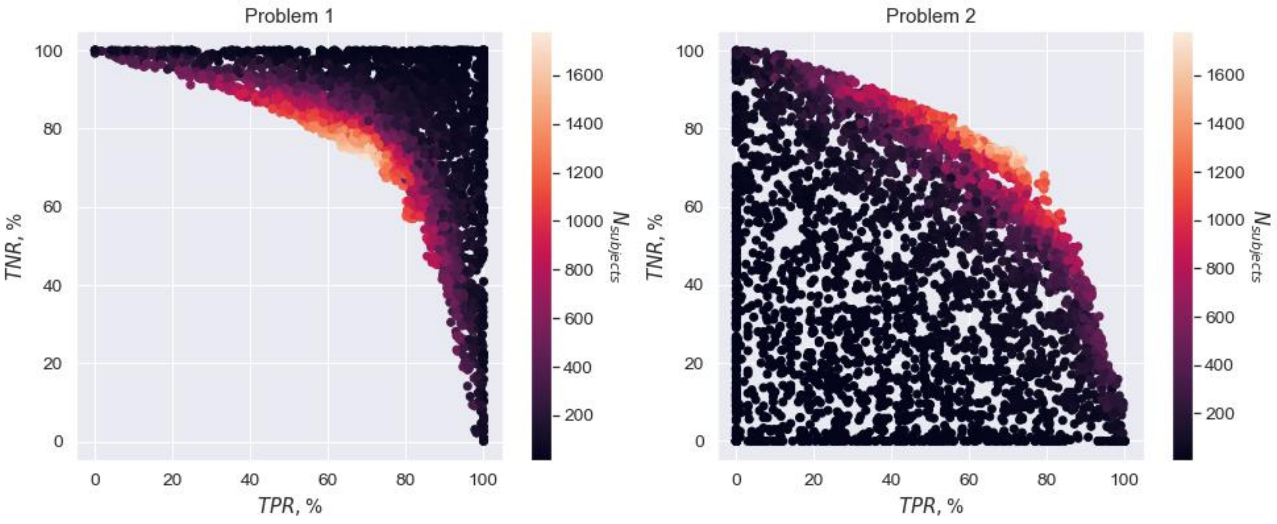

Figure S1: The initial sets of rules generated when solving Problems 1 and 2. Each point corresponds to one rule in the criterion space TPR-TNR, wherein a color indicates the number of subjects covered by the rule.

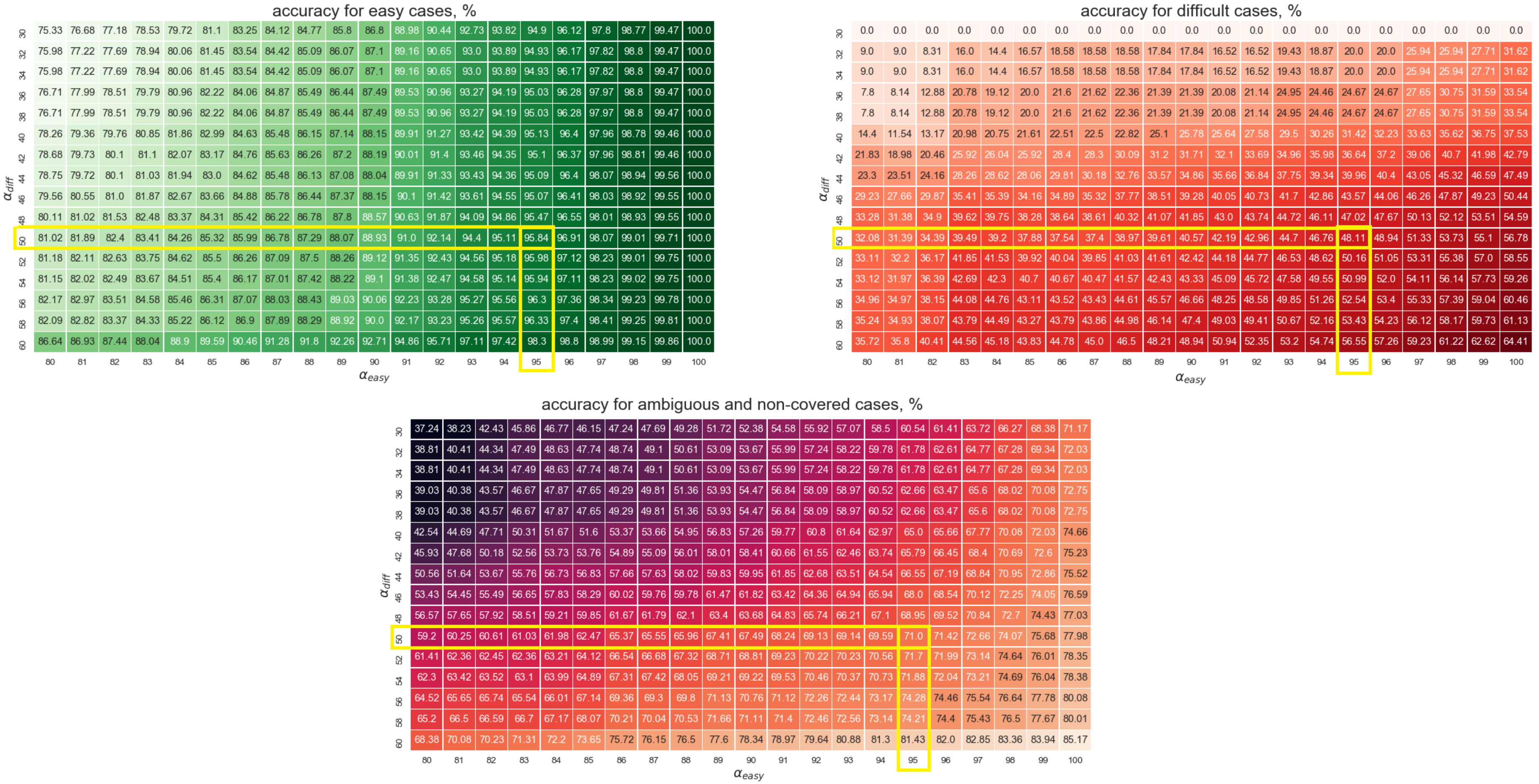

Figure S2: The overall accuracy for "easy", "difficult", "ambiguous" and "non-covered" subjects averaged over 50 runs of 5-fold cross-validation for different values of the thresholds α_{easy} and α_{diff}.

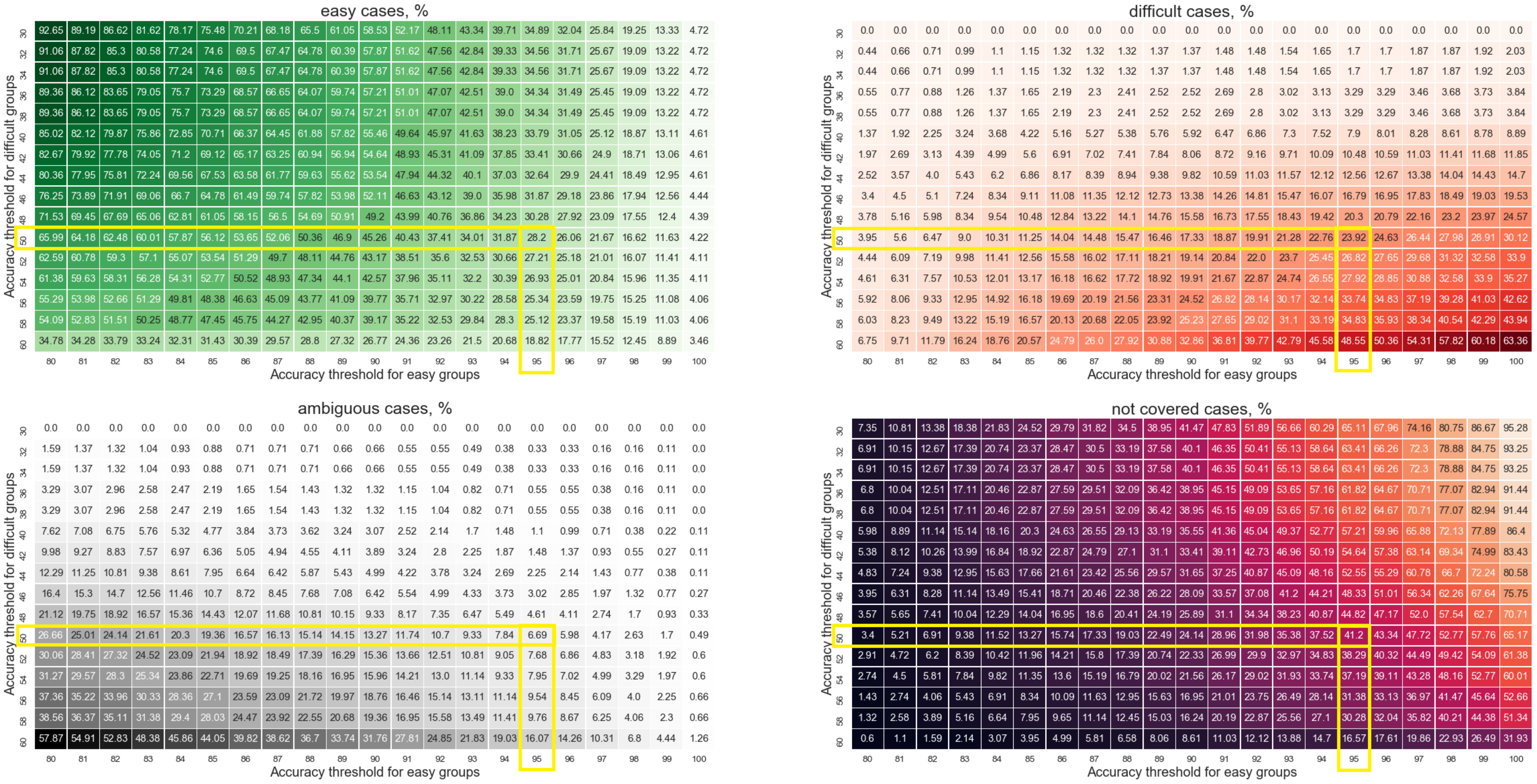

Figure S3: The percentage of “easy”, “difficult”, “ambiguous”, and “non-covered” subjects of the whole KIHD sample.
